# Laboratory Observations of Linkage of Preslip Zones Prior to Stick-Slip Instability

**DOI:** 10.3390/e20090629

**Published:** 2018-08-24

**Authors:** Yan-Qun Zhuo, Yanshuang Guo, Shunyun Chen, Yuntao Ji, Jin Ma

**Affiliations:** State Key Laboratory of Earthquake Dynamics, Institute of Geology, China Earthquake Administration, Jia 1 Huayanli, Chaoyang District, Beijing 100029, China

**Keywords:** earthquake precursor, fault displacement, normalized statistical parameter, digital image correlation method, spatial complexity of preslip

## Abstract

Field and experimental observations showed that preslip undergoes a transition from multiple to single preslip zones, which implies the existence of linkage of preslip zones before the fault instability. However, the observations of the linkage process, which is significant for understanding the mechanism of earthquake preparation, remains to be implemented due to the limitations of observation methods in previous studies. Detailed spatiotemporal evolutions of preslip were observed via a high-speed camera and a digital image correlation method in our experiments. The normalized length of preslip zones shows an increase trend while the normalized number of preslip zones (*N_N_*) shows an increase followed by a decrease trend, which indicate that the expansion of the preslip undergoes a transition from increase to linkage of the isolated preslip zones. The peak *N_N_* indicates the initiation of the linkage of preslip zones. Both the linkage of the preslip zones and the decrease in the normalized information entropy of fault displacement direction indicate the reduction of spatial complexity of preslip as the instability approaches. Furthermore, the influences of dynamic adjustment of stress along the fault and the interactions between the asperities and preslip on the spatial complexity of preslip were also observed and analyzed.

## 1. Introduction

The reduction of the disaster and losses caused by destructive earthquakes is a matter of concern to all humans. One fundamental but difficult solution is improving the accuracy of earthquake prediction based on the observations of earthquake precursors. Studying the physical process of earthquake preparation is the basis for understanding the mechanism of earthquake precursors. The motion of a seismogenic fault is jerky, which can be well described as “stick-slip” [1]. The heterogeneities in material composition, geometrical morphology, and mechanical properties of the fault result in the occurrence of its overall instability preceded by local instability. The local instability, which is caused by preslip in pre-existing faults, establishes a bridge from the stable “stick” state to the unstable “slip” state of the fault. The occurrence of preslip prior to the overall instability of a fault was found prevailing in laboratory [2,3], which is an important physical process during the earthquake preparation of faults. A large number of experiments have shown that the fault experiences an acceleration process in the preslip speed as well as the preslip zone expansion speed [3,4,5,6,7,8,9].

An earthquake nucleation model based on fracture mechanics or friction rate and state dependent constitutive relation [10,11] was proposed and stressed that the instability of a fault occurs when the preslip zone expands to a certain critical scale. The migration of foreshock sequences, which represented the propagation front of the preslip zone, was considered to be the earthquake nucleation process in field conditions [12]. Furthermore, the slow initial phase of seismic waves was also considered to be the earthquake nucleation process [13,14,15].

The concept of meta-instability state was proposed to describe the stage from the peak of the overall stress level of the fault system to the onset of the stick-slip instability based on laboratory observations [16], which revealed that the synergy of the relative physical fields of the fault is the characteristic of the meta-instability state [9,16,17,18,19]. The accelerating propagation of the earthquake sequences prior to the main shock was considered to be the entering of the meta-instability state [20]. The trend of the consistency of focal mechanism solutions between the foreshocks and the regional stress field of the main shock [21] was also consistent with the characteristic of the meta-instability state derived from experimental results [18].

The difference between the earthquake nucleation model and the meta-instability model is that the earthquake nucleation model emphasizes that the critical nucleation scale is the key to instability [10,11,22,23,24,25], which can be observed in the evolution of single preslip zone in homogeneous materials (e.g., resins) [6,7,26]. The meta-instability model emphasizes that the competitive evolution of multiple preslip zones leads to the transition of fault from isolated to synergetic activities [9,16,17,18,19], which was observed in heterogeneous materials (e.g., rocks) [8,9,16,17,18,19,20]. The earthquake nucleation model was proposed for preslip or pre-rupture zones, while the meta-instability model was proposed for the overall stress release of the sample. They are partially overlapped in the spatiotemporal evolution of preslip. Our previous studies [9,18,20] revealed that the preslip zones appear before the onset of the meta-instability stage. If the initial occurrence of preslip zones can be referred to as the beginning of the nucleation, then the nucleation process will include the meta-instability stage. The transition from multiple to single preslip zones prior to instability was usually observed due to the ubiquity of heterogeneity of natural as well as rock material simulated faults [8,9,16,17,18,19,20,27,28], which implies a process of linkage among the preslip zones. Such a linkage may drive the evolution of the preslip zones from quasi-static to quasi-dynamic propagation, which is the process of common concern between the earthquake nucleation and the meta-instability models. However, when the linkage occurs and how to identify the initiation of the linkage are still unclear due to the lack of effective observation of the detailed spatiotemporal evolution of preslip zones in previous studies. 

The spatial scale of the preslip zones can span multiple orders of magnitude during the earthquake preparation. As a result, high spatiotemporal sampling rates and continuous recording are required to observe this process. Traditional point-contact displacement gages or strain gages used in previous experiments cannot provide dense spatial observation of preslip. In this study, we used a high-speed camera combined with a digital image correlation (DIC) method to achieve dense spatial observations of preslip. By doing this, the linkage of preslip zones was observed and its initiation was identified by normalized statistical parameters.

## 2. Materials and Methods 

### 2.1. Experimental Design

**Figure 1:**
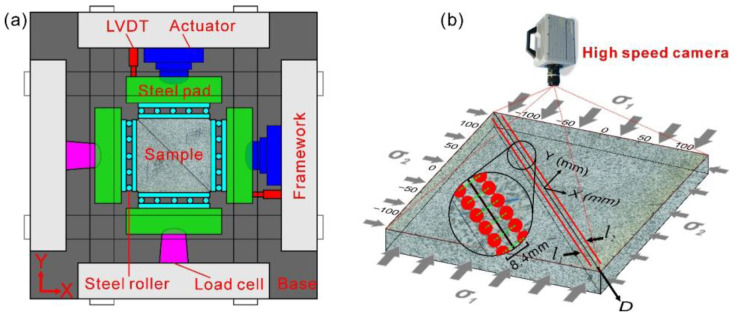
Schematic diagram of the experimental design. (**a**) The schematic diagram of the loading apparatus. LVDT: linear variable differential transformer. (**b**) The high-speed camera covered the entire upper sample surface. The *D* axis overlapped the fault trace. The center of the fault was the common origin of the axes *X*, *Y*, and *D*. The two red lines, denoted by *l_1_* and *l_2_* on both sides of the fault, were each composed of 1761 measuring points. The big circular illustration is an enlarged view of the small one showing the measurement points, in which each couple of red dots connected with an 8.4 mm-length green dashed auxiliary line represents a fault displacement sensor. The numbers of the fault displacement sensors increase from 1 to 1761 along the positive direction of *D*.

**Figure 2:**
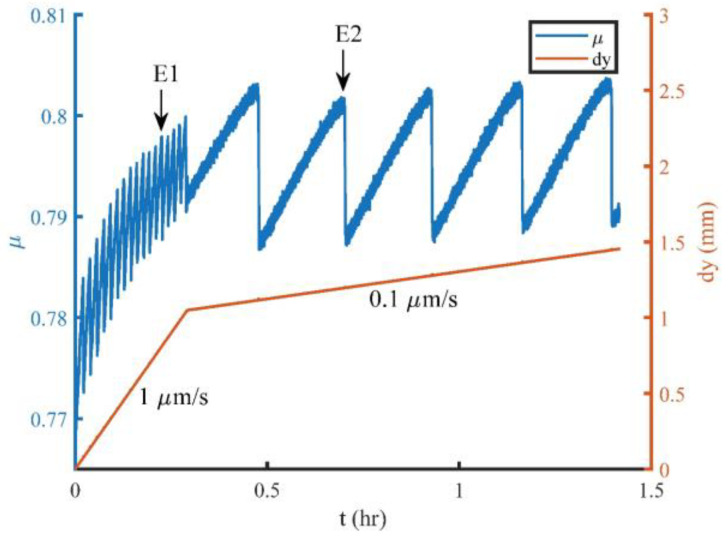
Variation of the friction coefficient (*μ*) and piston displacement along the *Y* axis (*dy*) with time (*t*). Stick-slip events labeled by E1 and E2 were analyzed in this study.

A granodiorite sample with an area of 300 mm × 300 mm and a thickness of 50 mm, which was used in our experiment, was cut through diagonally to form a straight fault before the experiment (Figure 1). The roughness of the fault surface is 100 μm before the experiment. During the experiment, the sample was loaded by a horizontal biaxial hydraulic servo-control machine (Figure 1a). A load from 0 to 5.0 MPa was applied synchronously on the *X* and *Y* axes of the sample as the first step. Then, the load along the *X* axis was held at a constant value of 5.0 MPa, while the load along the *Y* axis was changed to displacement rate servo-control at 1.0 μm/s followed by 0.1 μm/s to cause the dextral shearing and stick-slip motion of the sample fault. The variations of the friction coefficient (*μ*) and the piston displacement along the *Y* axis (*dy*) with time are shown in Figure 2. Each sudden drop of *μ* corresponds to a stick-slip event of the fault. Events E1 and E2 will be analyzed in this study. For the details about the mechanical parameters of the sample and loading system, please see our previous papers [9,18].

We used a high-speed camera (FASTCAM SA2, Photron, Tokyo, Japan) to capture images of the upper sample surface during Events E1 and E2 with acquisition durations of 2.8 s and 5.5 s, respectively. The sampling rate was 1000 frames per second. The resolution of each image was 2048 × 2048 pixels, and the actual size of each pixel corresponding to the surface of the sample was 149.5 × 149.5 μm^2^. It took 30 min for the computer to download the image data from the cache of the camera, during which the camera couldn’t record new images. Therefore, only Events E1 and E2 were recorded by the camera. The images were processed by a DIC method [9,18,29,30,31,32,33,34] to calculate the deformation of the sample. Two symmetric measurement lines (*l*_1_ and *l*_2_ in Figure 1a), each of which was composed of 1761 measurement points (pixels) and was 4.2 mm offset from the fault, were used to calculate the fault displacement and strain. Each couple of symmetric measurement points from the two measurement lines made up a fault displacement sensor, which meant that 1761 fault displacement sensors with spacing of 0.2 mm were used to measure the fault displacement. This dense distribution of fault displacement sensors enabled to observe the detailed spatial evolution of the preslip of the fault. The error of the fault displacement measurement was ±5 μm. For the details of the setting parameters of the DIC method, please see the Section 2.2.

### 2.2. Parameter Setting of DIC Method

The DIC method, which is a full-field and noncontact-type deformation measurement method based on pattern matching, has been widely used in experimental mechanics [9,18,32,33,34]. We also used this method to calculate the deformation field of the sample surface with the digital images of the original speckles of the sample surface obtained in the present experiment. The factors that influence the accuracy of the DIC measurement include the hardware system, the environment, the gray-level distribution in the image, the parameters set for calculation, etc. The systematic strain measurement error induced by self-heating of the cameras is remarkable and should be taken into account before measurement. According to a test of six cameras of four different types, a preheating period of at least 1–2 h is required to reduce the systematic strain measurement error [33]. In the present experiment, the preheating time was 3 h.

#### 2.2.1. Influence of the Subregion Size on the Correlation Coefficient

**Figure 3:**
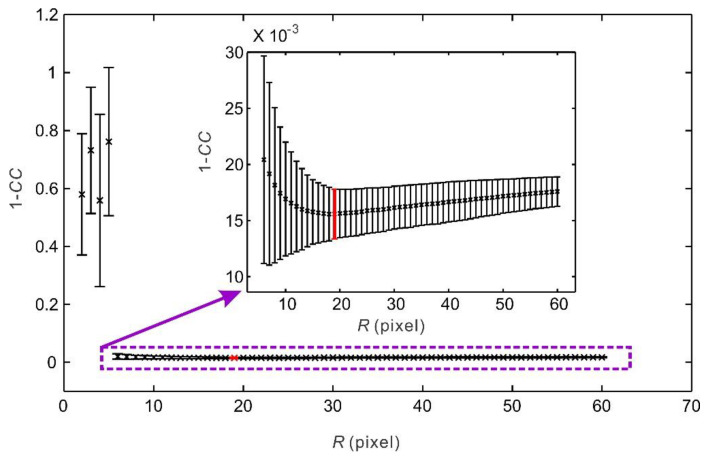
Influence of the subregion radius *R* on the correlation coefficient *CC* during the DIC calculation. Crosses denote the mean value of 1 − *CC*, error bars indicate the standard deviations, and the cross and error bar in red correspond to the optimal subregion radius.

A subregion centered at a point in the original image is chosen to calculate the correlation coefficient (*CC*) between the subregion and the moving subregion of the same size in the deformed image when the location of the point in the deformed image is searched. *CC* is an important parameter reflecting the subregion matching quality and the accuracy of the DIC calculation. *CC* ranges from 0 to 1 and increases as the subregion matching quality improves. It also depends on the subregion size (Figure 3). The average *CC* attains a peak value when the radius of a subregion *R* is 19 pixels. Although the standard deviation of *CC* decreases as *R* increases, the decrease of the standard deviation of *CC* is remarkable when *R* is less than 19 pixels but is inconspicuous when *R* is greater than 19 pixels. On the other hand, because the computational time and space filtering effect increase as the size of a subregion increases, a smaller subregion results in a better DIC calculation under the premise of a desirable *CC*. Thus, to obtain a high subregion matching quality and an accurate DIC calculation, 19 pixels will be the optimal value for *R*. Then, the size of the square subregion in this study is given by (2*R* + 1) × (2*R* + 1), namely, 39 × 39 pixels.

#### 2.2.2. Influence of the Subregion Size on the Fault Displacement Measurement

**Figure 4:**
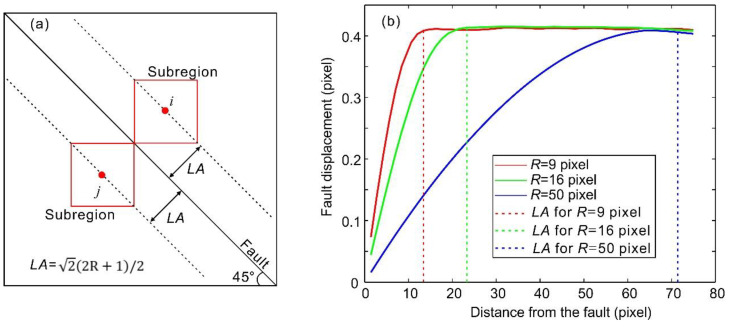
Influence of the fault influence length *LA* on the fault displacement measurement via the DIC method. (**a**) The red boxes denote the subregions of points *i* and *j*. The dashed lines at which the points *i* and *j* are located are offset from the fault at a distance of *LA*. The subregions will intersect the fault when their center points are located between the two dashed lines. (**b**) Changes in the fault displacement with the distance between the measurement point and the fault. The red, green, and blue lines indicate the calculation results for subregion radius *R* of 9, 16, and 50 pixels, respectively. The dashed vertical lines in red, green, and blue represent the values of *LA* for *R* of 9, 16, and 50 pixels, respectively.

Uncertainties will be generated during subregion matching when the fault trace and subregions intersect. The distance between a measurement point and the fault, which makes the fault intersect the subregion centered at the measurement point, is called the fault influence length and is denoted by *LA* (Figure 4a). As the fault is 45° oblique in the images, *LA* is half of the length of a subregion diagonal, namely, $LA=\sqrt{2}\left(2R+1\right)/2$. In order to explore the influence of the intersection between the fault trace and the subregions on the fault displacement measurement, three subregion radii (9, 16, and 50 pixels) were used to calculate the fault displacement during a stick–slip instability via the DIC method. Figure 4b shows the change in the fault displacement versus the distance between the fault and the measurement point for the three values of *R*. In Figure 4b, the common feature of the three lines is that the fault displacement increases followed by a steady period as the distance between the fault and the measurement point increases. The distinct difference between the three lines is that the distance between the fault and measurement point at which the fault displacement begins to be steady is larger with increasing *R*. This distance is equal to *LA*. As the fault displacement should be equal at the same position during the same stick-slip instability, this difference cannot reflect the true displacement of the fault. This is caused by the intersection between the fault and the subregion within *LA*. Therefore, the distance between the fault and the measurement point should not be less than *LA*. Thus, the distance between the fault and the measurement point should become larger with increasing *R*. Doing so will eliminate the intersection between the fault trace and the subregions. On the other hand, to accurately obtain instances of slip along the fault surface, the selected points used for the fault displacement measurement should be located as close as possible to the fault. Accordingly, the subregion size must be small to ensure that these measurement points are as close as possible to the fault. Therefore, for a chosen subregion size of *R*, the optimum distance from the measurement point to the fault is *LA*. Thus, *LA* will be 27.6 pixels when *R* is 19 pixels. As the length of one pixel is equal to 149.5 μm, the length of *LA* will be 4.2 mm, which is the distance between the fault and the measurement lines denoted by *l*_1_ and *l*_2_ in Figure 1b.

### 2.3. Definitions of Normalized Statistic Parameters

#### 2.3.1. Normalized Length of Preslip Zones

The locations of the fault where the dextral strike-slip component of the cumulative fault displacement is greater than 5 μm (the error of the fault displacement measurement) are considered as preslip zones, while other locations are considered as static zones. The length of a preslip or static zone can be calculated by the product of the number and spacing (0.2 mm) of the fault displacement sensors within the zone. By doing this, the total length of the preslip zones (*L_r_*) can be obtained. The normalized length of preslip zones (*L_N_*) [18] is defined as follows:

(1) $$L_{N}=L_{r}/L,$$

where *L* is the length of the fault. The value of *L_N_* is between 0 and 1. The higher its value is, the greater the proportion of the length of the fault is occupied by the preslip zones. Thus, *L_N_* indicates the level of the expansion of the fault preslip zones.

#### 2.3.2. Normalized Number of Isolated Preslip Zones

The total number of the fault displacement sensors is *N* (*N* = 1761), then the maximum number of the isolated preslip zones will be *N_m_* = *N*/2 (when *N* is an even number) or *N_m_* = (*N* + 1)/2 (when *N* is an odd number). *N_m_* means that the length of each preslip/static zone is the same and equal to the minimum value (0.2 mm) and the preslip and static zones are located alternatively. Therefore, the actual number of the isolated preslip zones (*N_r_*) is not greater than *N_m_*. The normalized number of isolated preslip zones is defined as follows:

(2) $$N_{N}=N_{r}/N_{m}.$$

From Equation (2), the value of *N_N_* is between 0 and 1, which represents the degree of dispersion of the preslip distribution along the fault. The smaller the *N_N_* is, the more concentrated the preslip zones distribute along the fault.

#### 2.3.3. Normalized Information Entropy of Fault Displacement Direction

The normalized information entropy of fault displacement direction (*S_N_*) is used to describe the dispersion degree of the displacement direction of each part of the fault based on the concept of Shannon entropy [35]. *S_N_* is defined as follows [18]:

(3) $$S_{N}=-\sum_{i=1}^{Q}p\left(i\right){log}_{Q}p\left(i\right),$$

where *Q* is the total number of divided direction intervals within 360° (direction interval of 5° was used in this study and *Q* = 72 accordingly). The use of *Q* as the base of the logarithm makes *S_N_* normalized. *p*(*i*) is the probability that the direction of the fault displacement measured by the fault displacement sensors within the *i*th direction interval. *p*(*i*) is defined as follows [18]:

(4) $$p\left(i\right)=N_{i}/N,$$

where *N_i_* is the number of fault displacement sensors with fault displacement direction within the *i-*th direction interval; *N* is the total number of fault displacement sensors (*N* = 1761). Condition of *p*(*i*) = 0 will be omitted when Equation 3 is calculated. The value of *S_N_* is between 0 and 1. The smaller the *S_N_* is, the more concentrated the distribution of the fault displacement directions is.

## 3. Results

### 3.1. Identify Linkage of Preslip Zones by the Peak N_N_

**Figure 5:**
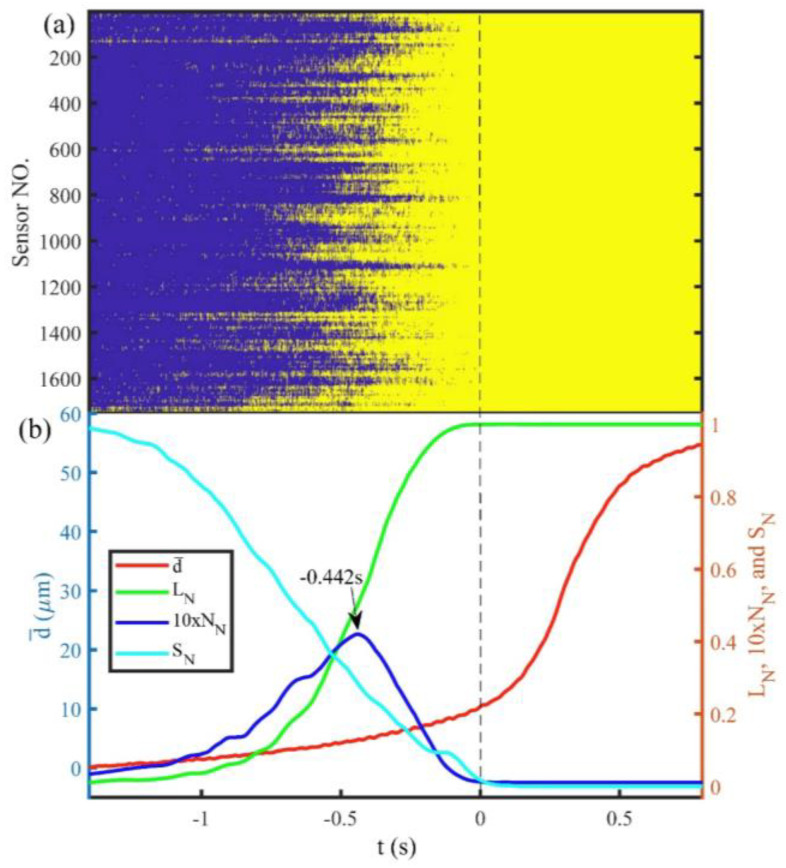
Spatiotemporal evolution of preslip zones and variations of $\bar{d}$, *L_N_*, *N_N_*, and *S_N_* with time during Event E1. (**a**) Spatiotemporal evolution of preslip zones. The preslip zones are shown in yellow. The blue areas represent static zones. The black dashed line in zero time represents the onset of the stick-slip instability of Event E1. The sensor number increases along the positive direction of axis *D*. (**b**) The black arrow denotes the peak value of *N_N_* and its time. $\bar{d}$: the average cumulative fault slip. *L_N_*: the normalized length of preslip zones. *N_N_*: the normalized number of isolated preslip zones. *S_N_*: the normalized information entropy of fault displacement direction.

**Figure 6:**
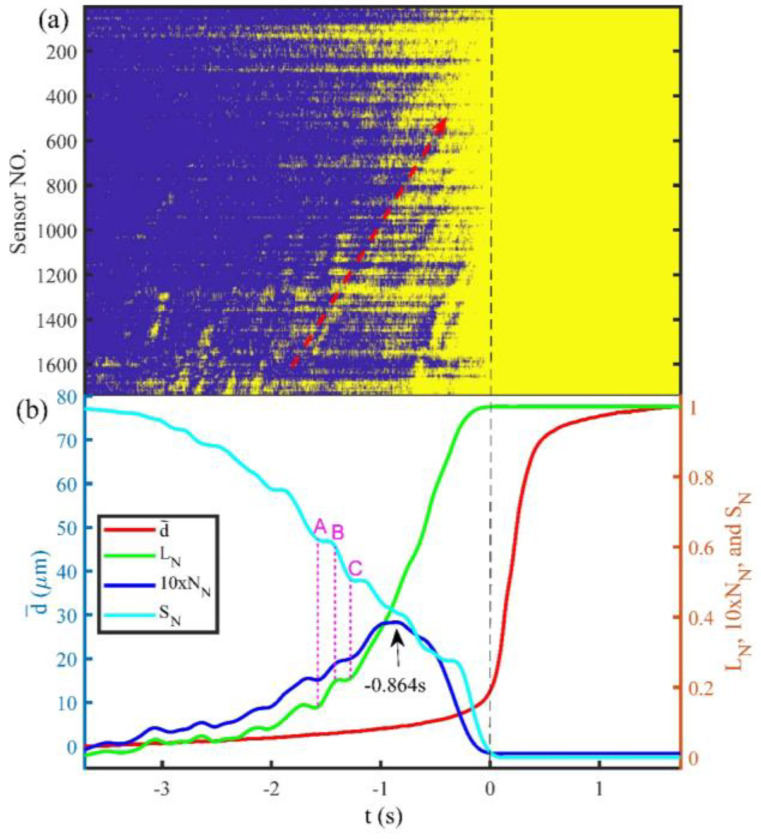
Spatiotemporal evolution of preslip zones and variations of $\bar{d}$, *L_N_*, *N_N_*, and *S_N_* with time during Event E2. (**a**) The red dashed arrow denotes a migration path of preslip zones. (**b**) The pink dashed lines denoted by A, B, and C labeled the two intervals, in which the normalized statistical parameters fluctuate. $\bar{d}$: the average cumulative fault slip. *L_N_*: the normalized length of preslip zones. *N_N_*: the normalized number of isolated preslip zones. *S_N_*: the normalized information entropy of fault displacement direction. Other labels are the same as shown in Figure 5.

Figure 5 and Figure 6 show the spatiotemporal evolution of the preslip zones and changes of the normalized statistical parameters with time during the Events E1 and E2, respectively. The zero time represents the onset of the stick-slip instabilities. The yellow areas in Figure 5a and Figure 6a represent the preslip zones, while the blue areas represent the static zones. The preslip zones expand independently prior to the onset of Event E1, but they migrate along the fault (the red dashed arrow represents the migrating direction) as they expand before the onset of Event E2. Figure 5b and Figure 6b are the changes of the average cumulative fault slip ($\bar{d}$), *L_N_*, *N_N_*, and *S_N_* with time during the Events E1 and E2, respectively. *N_N_* is displayed after magnified 10 times to ensure the change of it is visible. As shown in Figure 5b and Figure 6b, before the onset of the stick-slip instability, $\bar{d}$ accelerates continuously, *L_N_* shows an increasing trend, *N_N_* shows a transitional trend from increase to decrease (the peak values of *N_N_* are −0.442 s and −0.864 s prior to Events E1 and E2, respectively), and *S_N_* shows a decreasing trend. The evolutions of *L_N_* and *N_N_* indicate that the expansion of the preslip zones along the fault begins with an increase in the number of isolated preslip zones but changes to the linkage of the isolated preslip zones. The peak value of *N_N_* indicates that linkage among the preslip zones begins to be a dominant factor controlling the evolution of preslip. 

Thus, the initiation of linkage of preslip zones can be identified by the peak value of *N_N_*. The decrease in *S_N_* indicates that the displacement direction of each part of the fault gradually tends to be consistent. In addition, the changes of the normalized statistical parameters have the following common features: the rate of the increase of *L_N_* and the rates of the decline of *N_N_* and *S_N_* become slower as the instability approaches.

### 3.2. Fluctuation of the Normalized Statistical Parameters

The preslip zones exhibit a migrating propagation in Event E2 compared with Event E1. Accordingly, the evolutions of the normalized statistical parameters show more conspicuous fluctuations in Event E2 than that in Event E1 before the *N_N_* reaches its peak values. The fluctuations of the normalized statistical parameters appear as a pause or a reverse in their increase or decrease trends (Figure 6b). In addition, the three normalized statistical parameters exhibit different fluctuations. As shown in Figure 6b, *L_N_* and *N_N_* show a steady rise that are consistent with their general trends during the AB period, while *S_N_* appears to a pause that is inconsistent with its general decreasing trend. However, during the BC period, *L_N_* and *N_N_* show a pause or decline that are inconsistent with their general increasing trends, while *S_N_* shows a steady reduction that is consistent with its overall trend. These differences are contributed to the continuous acceleration of $\bar{d}$ and the double types of propagation of the preslip zones in Event E2. In the AB phase, the propagation of the preslip zones is dominated by the independent expansion. As a result, *L_N_* and *N_N_* increase due to the generation of new preslip zones. However, the directions of the fault displacement within the new preslip zones are dispersed due to the comparable values of the strike-slip and normal-slip components, which subsequently cause the pause in *S_N_*. In the BC phase, the propagation of preslip zones is dominated by migration, which does not enlarge the preslip range. Accordingly, *L_N_* and *N_N_* are unchanged or reduced slightly. However, the continuous accelerating $\bar{d}$ within the unchanged preslip range means the acceleration of preslip, which increases the dextral strike-slip component of the fault displacement in the preslip zones and accordingly reduces *S_N_*. 

### 3.3. Migration of Localized Strain Bands Controls the Migration of Preslip Zones

**Figure 7:**
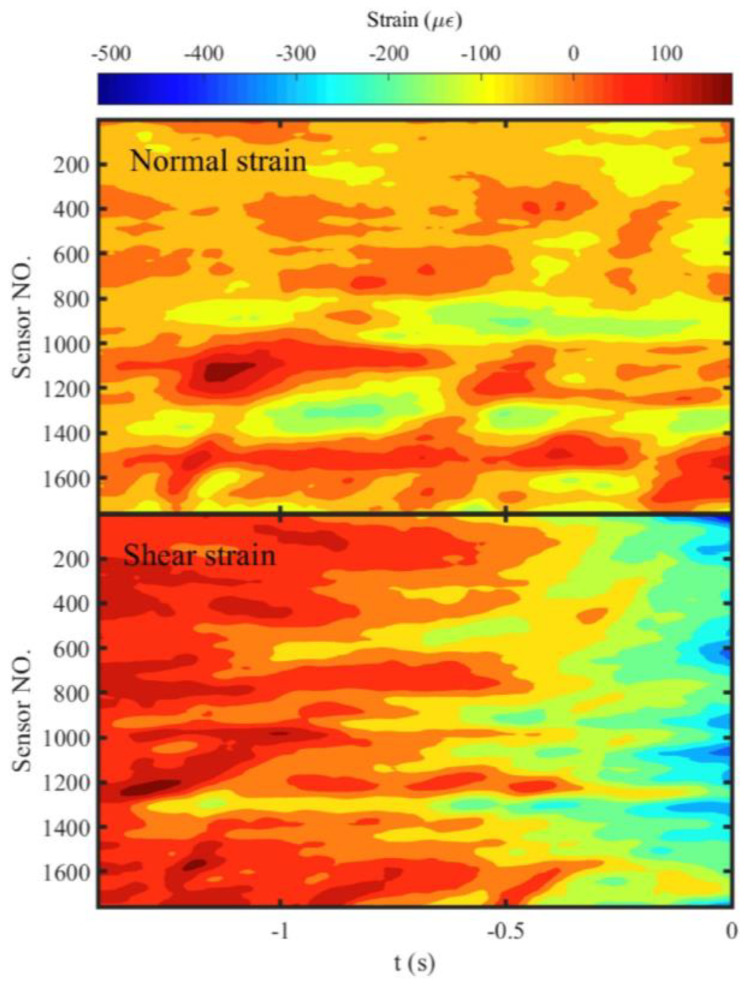
The spatiotemporal evolution of normal and shear strains along the fault prior to Event E1. The negative values of the strain indicate the release of strain. The zero time indicates the onset of the stick-slip instability. The sensor number increases along the positive direction of axis *D*.

**Figure 8:**
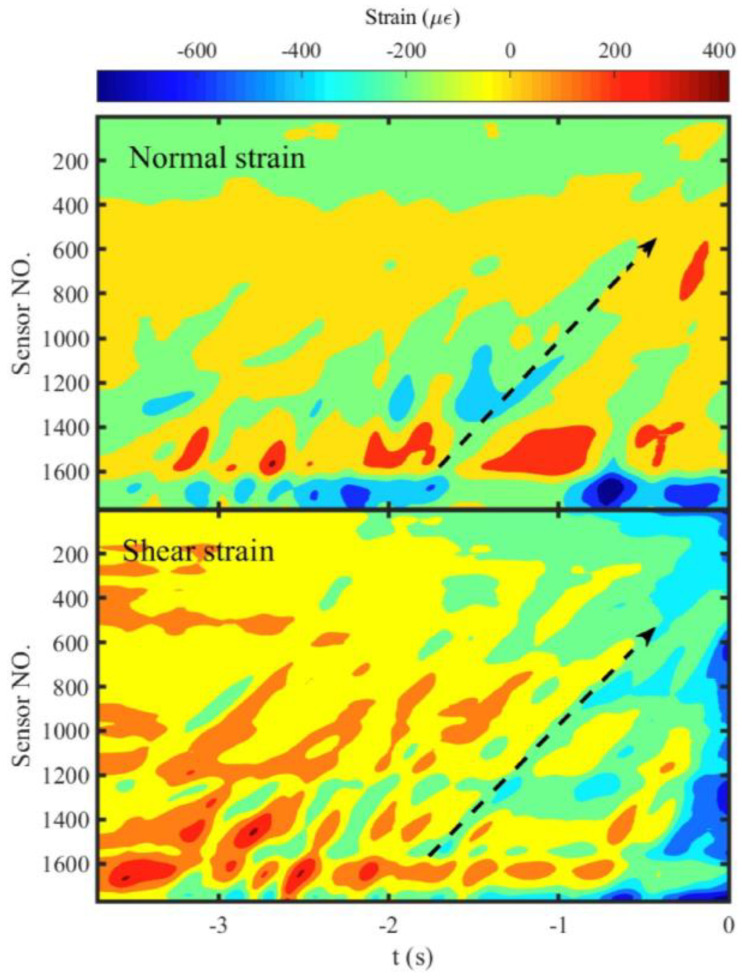
The spatiotemporal evolution of normal and shear strains along the fault prior to Event E2. The black dashed arrows, which have the same spatiotemporal locations as the red dashed arrow in Figure 6a, indicate the migration path of a localized strain band. Other labels are the same as shown in Figure 7.

The normal and shear strains along the fault were calculated with the measurement points in *l_2_*. The duration before the stick-slip instability was drawn in Figure 7 and Figure 8 to ensure the visible of the process which will be too small to be invisible compared with the large change during the instability. The spatiotemporal evolutions of the normal and shear strains prior to Events E1 and E2 are shown in Figure 7 and Figure 8, respectively. The localized strain bands can be identified in the two events. The difference is that the localized strain bands show parallel to the time axis in Event E1, which is consistent with the independent expansion of the preslip zones. However, they are oblique to the time axis in Event E2. The black dashed arrows in Figure 8 have the same spatiotemporal locations as that shown in Figure 6a, which indicates the spatiotemporal correlation between the migrations of the preslip zones and localized strain bands. Therefore, the migration of the preslip zones is caused by the migration of the localized strain bands that represent the dynamic adjustment of stress along the fault. 

## 4. Discussion

Synergistic activity along faults was observed in field conditions [21]. In our previous study, the spatiotemporal evolution of the fault displacement in the middle part of the fault was observed and the decrease of *S_N_* was revealed, which indicated the synergistic activity of the fault displacement before the instability [18]. The similar process was again observed in this study with fault displacement observations covering the entire fault, which showed the evidence for synergistic activity along the entire fault prior to its instability. The use of the evolutions of *L_N_* and *N_N_* revealed that the linkage of the isolated preslip zones is the mechanism of the transition from multiple preslip zones to single preslip zone prior to the stick-slip instability. The linkage of the preslip zones initiates at the point where *N_N_* reaches its peak value and enhances the connection of different sliding segments along the fault. As a result, the activities of different parts of the fault tends to be consistent and the spatial complexity of preslip reduces, which may be the mechanism of the synergistic activities of faults observed in field or laboratory conditions.

The density of spatial distribution of sensors plays crucial role in uncovering the evolution of preslip in the laboratory. The use of one displacement gage mounted near a simulated fault could detect stick–slip preceded by preslip, which could not be readily observed at the specimen endcaps [2]. However, the use of multiple strain/slip gages can detect the propagation of a single preslip zone [3,4,5]. In recent years, multiple preslip zones were detected by using multiple types of gages (piezoelectric sensors, strain gages, and slip sensors) mounted on both the top and bottom sample surfaces [8], dense distribution of strain gages [20], or very dense distribution of fault displacement sensors via the DIC method [9,18]. The very dense distribution of the fault displacement sensors via the DIC method in this study again enable to obtain a detailed spatial distribution of the preslip zones that show a transition from randomness to concentration due to the linkage of them. These indicate the dependence of how much we know about the evolution of preslip on the development of the observation technology or methods.

Experimental studies have observed the interaction between the preslip and asperities, which revealed that the asperities can obstruct and promote the preslip before and after their failure, respectively [8,36]. The stability of the fault is actually supported by a few strong asperities [37,38]. The successive failure of these asperities causes a large amount of stress to accumulate on the last static asperity, which means that more strain energy needs to be accumulated to break the asperity. Therefore, the last static asperity will have an obstructive effect on the preslip behavior before its failure, which leads to the slower rates of increase of *L_N_* and decreases of *N_N_* and *S_N_* prior to the stick-slip instability as shown in Figure 5b and Figure 6b.

The migration of localized strain bands along the fault (as shown in Figure 8) was similar in other experiments previously reported in our conference paper [39], the origin of which is still not clear (it may be caused by a complicated interactions between preslip and deformation of asperities, or the additional load generated by the press system under certain conditions). However, its effect on the preslip is obvious. The spatiotemporal correlation between the migration of the localized strain bands and the preslip zones shown in Figure 6a and Figure 8 indicates that the dynamic adjustment of the stress on the fault controls the spatiotemporal variation of the preslip zones and leads to the fluctuations of *L_N_*, *N_N_*, and *S_N_* shown in Figure 6b. The fluctuations will probably bring uncertainty to the real-time identification of the peak *N_N_* which indicates the approach of the impending instability. Therefore, future study on the origin of the dynamic stress adjustment will be of great significance for reducing the uncertainty. Furthermore, such a future study may also implement the new method of natural time analysis which has been applied to laboratory measurements [40] and dynamical models [41,42] associated with stick-slip processes as well as to real seismic data of Japan [43,44], California [45], etc., in order to identify the approach of the system to the critical point. Notably, natural time has been recently used as a basis for a new method of estimation of seismic risk by Turcotte and coworkers [46,47,48,49].

## 5. Conclusions

The detailed spatiotemporal evolution of preslip zones was observed in this study, based on which three normalized statistical parameters were defined to quantitatively describe the evolution of preslip. The increase in *L_N_* and the transition from increase to decrease in *N_N_* indicate that the expansion of the preslip zones along the fault begins with an increase in the number of isolated preslip zones but changes to the linkage of the isolated preslip zones. The peak *N_N_* is an identifier of the initiation of linkage among preslip zones. The linkage of the preslip zones and the decrease in *S_N_* indicate the reduction of the spatial complexity of preslip, which is subjected to fluctuation by the migration of the localized strain bands controlling the migration of the preslip zones and is slowed down by the interactions between the preslip and asperities as the instability approaches.
